# Choosing care homes as the least preferred place to die: a cross-national survey of public preferences in seven European countries

**DOI:** 10.1186/1472-684X-13-48

**Published:** 2014-10-23

**Authors:** Natalia Calanzani, Katrien Moens, Joachim Cohen, Irene J Higginson, Richard Harding, Luc Deliens, Franco Toscani, Pedro L Ferreira, Claudia Bausewein, Barbara A Daveson, Marjolein Gysels, Lucas Ceulemans, Barbara Gomes, on behalf of Project PRISMA

**Affiliations:** Department of Palliative Care, Policy & Rehabilitation, Cicely Saunders Institute, King’s College London, London, SE5 9PJ UK; Centre for Population Health Sciences, University of Edinburgh, Teviot Place, Doorway 1, Edinburgh, EH8 9AG UK; End-of-Life Care Research Group, Vrije Universiteit Brussel (VUB) & Ghent University, Laarbeeklaan 103, 1090 Brussels, Belgium; VU University Medical Center, EMGO Institute for Health & Care Research, Palliative Care Center of Expertise and Department of Public & Occupational Health, P.O. Box 7057, 1007 MB Amsterdam, The Netherlands; Fondazione Lino Maestroni - ONLUS, Istituto di Ricerca in Medicina Palliativa, Via Palestro, 1, 26100 Cremona, Italy; Centre for Health Studies and Research (CEISUC), Faculty of Economics, University of Coimbra, Av Dias da Silva 165, 3004-512 Coimbra, Portugal; Department of Palliative Medicine, Munich University Hospital, Campus Großhadern, Marchioninistr, 15, Munich, Germany; Centre for Social Science and Global Health, University of Amsterdam, O.Z. Achterburgwal 185, 1012 DK Amsterdam, The Netherlands; Barcelona Centre for International Health Research (CRESIB – Hospital Clínic – Universitat de Barcelona), Rosselló 132, SA 1ª, Barcelona, 08036 Spain; University Antwerp Belgium, Campus Drie Eiken, D.R.307, Universiteitsplein 1, 2610 Wilrijk, Antwerp, Antwerp Belgium

**Keywords:** Public health, Europe, Health care surveys, Care homes, Preferences, Palliative care

## Abstract

**Background:**

Care homes are increasingly becoming places where people spend the final stages of their lives and eventually die. This trend is expected to continue due to population ageing, yet little is known about public preferences regarding this setting. As part of a larger study examining preferences and priorities for end of life care, we investigated the extent to which care homes are chosen as the least preferred place of death, and the factors associated with this negative preference.

**Methods:**

We conducted a cross-sectional telephone survey among 9,344 adults from random private households in England, Flanders, Germany, Italy, the Netherlands, Portugal and Spain. We asked participants where they would least prefer to die in a situation of serious illness with less than one year to live. Multivariate binary logistic regressions were used to identify factors associated with choosing care homes as the least preferred place of death in each country.

**Results:**

Care homes were the most frequently mentioned least preferred place of death in the Netherlands (41.5%), Italy and Spain (both 36.7%) and the second most frequent in England (28.0%), Portugal (25.8%), Germany (23.7%) and Flanders (18.9%). Only two factors had a similar and significant effect on the least preferred place of death in more than one country. In Germany and the Netherlands those doing housework were less likely to choose care homes as their least preferred place (AOR 0.72; 95% CI:0.54-0.96 and AOR 0.68; 95% CI:0.52-0.90 respectively), while those born in the country where the survey took place were more likely to choose care homes (AOR 1.77; 95% CI:1.05-2.99 and AOR 1.74; 95% CI:1.03-2.95 respectively). Experiences of serious illness, death and dying were not associated with the preference.

**Conclusions:**

Our results suggest it might be difficult to promote care homes as a good place to die. This is an urgent research area in order to meet needs and preferences of a growing number of older people with chronic, debilitating conditions across Europe. From a research perspective and in order to allow people to be cared for and die where they wish, our findings highlight the need to build more in depth evidence on reasons underlying this negative preference.

**Electronic supplementary material:**

The online version of this article (doi:10.1186/1472-684X-13-48) contains supplementary material, which is available to authorized users.

## Background

In the current European context of an ageing population, incidence of cancer and chronic, debilitating conditions such as dementia is a growing concern [1]. Specialised geriatric, palliative and social care is required, especially for those needing institutionalised care [2].

Residential and nursing care homes, along with other long-term care settings have increasingly become places where people spend the final stages of their lives [3, 4]. In England and Wales, a fifth (20.7%) of all deaths in 2012 took place in a care home [5]. A shift from hospital to care home deaths has been observed in Belgium and the Netherlands from 1998 to 2007, especially for patients with dementia [6]. These shifts are happening despite the fact that most people would prefer a home death [7] and show a strong wish to keep direct control of their lives, remaining independent for as long as possible [8].

Enabling people to make genuine choices about the care they receive towards the end of life is a well-recognised value in palliative care [9]. Place of death has also been recommended as a quality indicator of palliative care [10]; in the UK death in the usual place of residence (which may be a care home) is a key performance indicator for those providing end of life care [11]. Across Europe, most people die at an older age [12], which makes listening to their preferences regarding care, treatment and place of care and death especially important.

Although evidence is clear on the fact that most people would prefer to die at home [7], less is known about places where people would not wish to spend the end of their lives. In Europe there are reports of care homes having a poor public image, and concerns about quality of care, abuse by staff and very high costs of care [4, 13–15]. In the context of growing need for care home beds for older people likely to require assistance for long-term chronic conditions [1], an increasing number of deaths taking place in care homes, and the need to help people meet their care preferences at the end of life, more information is needed about the general population’s views on reaching the end of life in these facilities.

Cross-national comparisons show that European countries have different availability of care home beds and proportions of care home deaths [6, 16, 17]. The service provision in care homes (including palliative care provision and availability) and source of funding are also diverse [3, 4, 13, 18–20]. Therefore, this study aimed at a cross-national investigation of the extent to which care homes are chosen as the least preferred place of death, and the factors associated with this negative preference.

## Methods

### Study design

We conducted a population-based telephone survey across seven European countries as part of a European Commission project funded by the 7^th^ Framework called “Reflecting the Positive diveRsities of European prIorities for reSearch and Measurement in end of life cAre (PRISMA)”, a collaborative with the aim to co-ordinate high-quality research into end of life cancer care [21]. The survey covered Flanders, England, Germany, Italy, Netherlands, Spain and Portugal.

### Measurement

The survey was grounded on a theoretical model of preferences for place of death which describes preferences as a result of three factors: facilitating circumstances (such as general health, education and income), prior experiences (e.g. experiencing personal illness or caring for someone who is dying) and personal values (such as the importance attributed to dying in the preferred place). More information on the model is available elsewhere [22–24].

Residential and nursing care homes refer to institutional settings providing care for older people who live in these settings continuously during an undefined period of time. The care provided includes assistance with activities of daily living (ADLs), nursing and medical care. The level of care varies according to the residents’ dependency levels (more to less dependent), and different countries have different institutions in place to meet the needs of different patients [19].

The questionnaire included 28 questions on preferences, personal values related to end of life care (questions 1–10), socio-demographic questions including questions on general health and disability (questions 11–23) and experiences of serious illness, death and dying (questions 24–27). The questionnaire is available online in Additional file 1.

We asked participants *“In a situation of serious illness like cancer with less than one year to live…Where do you think you would prefer to die if circumstances allowed you to choose?”.* This was followed by the question: “*So which of these do you think you would least prefer if circumstances allowed you to choose?” Answer options were: “in your own home”, “in the home of a relative or friend”, “in a hospice or palliative care unit – places with specialised care and beds for dying patients”, “in a hospital – but not in a palliative care unit”, “in a care home” and “somewhere else”.* The term “care home” was phrased differently across countries and adapted according to language and service availability in order to be understood by all interviewees and for the data to remain comparable cross-nationally.

Original questions asked in each country are available online in Additional file 2. In England and the Netherlands both “nursing home” and “residential home” were available as answer options, later merged for analysis into a single variable response of “care homes”. Details on the questionnaire development and piloting using cognitive interviewing are available [24, 25]. Information about power calculations, data on the most preferred place of death and analyses of the other survey questions can be found elsewhere [24, 26–30].

Participants include both those who completed (more than 90% of questions asked, regardless of the answers to the questions) and partially completed the interviews (more than 60% but less than 90% of the questions asked). Interviews that did not reach 60% completion were classified as “break-offs” and were not included in the analysis. Response rate was calculated by dividing all complete and partial responses (numerator) by the sum of all the calls which identified an eligible participant (these included complete and partial responses, break-offs and requests to call back which were not completed) and refusals to take part (the denominator). Interviewers entered answers into a database with missing data checks at entry; this was then imported into SPSS version 18.0 for analysis.

### Participants

We invited individuals aged ≥16 residing in a private household to participate in a telephone interview. All households were selected by using random digit dialling (RDD). If an eligible individual refused to participate no substitution was allowed in the same household. Participants were excluded if not capable of hearing or understanding the information provided, incapable of providing informed consent (verified by the interviewers) or had poor language skills (of the country’s dominant language).

### Statistical analysis

We described the sample and crude percentages for the least preferred place of death, used χ2 tests to compare crude percentages for categorical data, Mann–Whitney tests to check for differences in ordinal data and t-test to analyse differences in age (as this was normally distributed). We analysed all variables in the questionnaire previously identified as associated with preferences and priorities according to the theoretical model postulating that preferences result from three groups of factors: facilitating circumstances, prior experiences and personal values [24]. A list of variables tested in bivariate analysis is available online in Additional file 3.

We conducted multivariate binary logistic regression analysis for each country separately with ‘care home’ (vs. ‘all other responses’) as least preferred place of death as the dependent variable. We entered country-specific factors associated with choosing care homes as the least preferred place of death in the bivariate analysis as the independent variables (p ≤0.05). We also forced entry for variables that were significant at least overall (data for all countries shown together) or in two different countries, as long as the direction of effect was consistent across all countries. These variables were entered in order to identify cross-national covariates and confounders. Tests were two-tailed, p ≤ 0.01 was deemed significant in the final regression models; we excluded all cases with missing data. We also evaluated how the models fit the observed data using receiver operating characteristic (ROC) curve, Nagelkerke R2 and Hosmer-Lemeshow goodness-of-fit test.

### Ethics

The study was approved by the King’s College research ethics committee (ref: BDM/08/09-48); country-specific ethical approval was also obtained in the Netherlands (Medical Ethics Committee at the VU Medical Centre; ref: 2009/342). Additional ethical approvals were requested but not required in Germany (Working Group of Medical Ethics Committee), Spain (Ethics Committee at the Hospital Clínic in Barcelona) and Italy (Tuscany Regional Commission of Bioethics). This was due to the nature of the study and type of data collected, i.e. no physical intervention, no research with patients or search for patient files (Germany and Spain); and no competence regarding public opinion polls over the telephone (Italy). Further ethical approvals were not needed in Portugal and Belgium; in both countries local data protection agencies (the National Commission for Data Protection in Portugal and the Privacy Commission in Belgium) were notified about the study.

## Results

### Sample characteristics

A total of 9,344 people from 45,242 randomly selected private households with a known eligible person agreed to participate in the study giving a response rate of 21% (Table 1). The responses corresponded to 9,304 complete and 40 partial interviews. Response was highest in Germany (29%), followed by Portugal (28%), Spain (21%), Italy (21%), England (21%), Flanders (16%) and the Netherlands (16%). A total of 2,835 people broke-off before reaching 60% of the questionnaire, 2,342 requests to call back were not completed and 30,721 people refused to take part. A thorough description of reasons for refusal can be found elsewhere [22]; main specified reasons for refusal were lack of interest (59%) and lack of time (17%). The interviews took on average 15.4 minutes to complete (range 3 to 91 minutes).

**Table 1:** **Least preferred place of death and demographics of participants by country (N = 9,344)**

Variables ^*, †^	EN ***N*** = 1,351	FL ***N*** = 1,269	DE ***N*** = 1,363	IT ***N*** = 1,352	NL ***N*** = 1,356	PT ***N*** = 1,286	ES ***N*** = 1,367	All ***N*** = 9,344
	n(%)	n(%)	n(%)	n(%)	n(%)	n(%)	n(%)	n(%)
**Least preferred place of death:**								
Own home	104 (8.0)	128 (10.7)	93 (7.1)	103 (8.3)	56 (4.3)	257 (21.8)	138 (11.2)	879 (10.0)
Home of a relative or friend	185 (14.3)	244 (20.4)	122 (9.4)	129 (10.4)	132 (10.1)	171 (14.5)	233 (18.9)	1,216 (13.9)
Hospice or palliative care unit	95 (7.3)	101 (8.5)	104 (8.0)	201 (16.2)	83 (6.4)	58 (4.9)	101 (8.2)	743 (8.5)
Hospital – but not palliative care unit	536 (41.5)	464 (38.8)	657 (50.5)	329 (26.4)	460 (35.2)	346 (29.4)	284 (23.0)	3,076 (35.1)
Care home	362 (28.0)	226 (18.9)	308 (23.7)	457 (36.7)	542 (41.5)	304 (25.8)	454 (36.7)	2,653 (30.3)
Elsewhere	11 (0.9)	32 (2.7)	17 (1.3)	25 (2.0)	32 (2.5)	42 (3.6)	26 (2.1)	185 (2.1)
**Demographics:**								
Age								
Mean in years (SD)	54.2 (16.3)	52.2 (14.3)	47.1 (15.7)	48.7 (15.9)	54.5 (14.6)	50.1 (16.9)	48.1 (16.5)	50.7 (16.0)
16-29	107 (8.0)	88 (7.5)	213 (15.8)	177 (15.0)	61 (4.7)	169 (13.8)	204 (15.4)	1,019 (11.5)
30-39	151 (11.3)	119 (10.2)	197 (14.6)	166 (14.1)	126 (9.7)	176 (14.4)	213 (16.1)	1,148 (12.9)
40-49	255 (19.1)	261 (22.3)	361 (26.8)	241 (20.4)	289 (22.3)	231 (18.9)	279 (21.0)	1,917 (21.6)
50-59	258 (19.4)	315 (27.0)	273 (20.3)	272 (23.1)	313 (24.2)	246 (20.1)	294 (22.2)	1,971 (22.2)
60-69	317 (23.8)	256 (21.9)	184 (13.7)	209 (17.7)	306 (23.6)	226 (18.5)	198 (14.9)	1,696 (19.1)
70+	244 (18.3)	129 (11.0)	119 (8.8)	115 (9.7)	199 (15.4)	175 (14.3)	139 (10.5)	1,120 (12.6)
Country of birth								
Born in country	1,201 (89.0)	1,205 (95.0)	1,233 (90.6)	1,298 (96.1)	1,275 (94.2)	1,168 (90.8)	1,275 (93.4)	8,655 (92.7)
Gender								
Female	863 (63.9)	832 (65.6)	790 (58.0)	974 (72.0)	891 (65.8)	893 (69.4)	935 (68.4)	6,178 (66.1)
Living arrangements								
Living alone	325 (24.2)	197 (15.6)	281 (20.8)	142 (10.5)	294 (21.8)	136 (10.6)	156 (11.5)	1,531 (16.5)
Marital status								
Married or with a partner	822 (61.3)	951 (75.7)	784 (58.1)	860 (63.8)	932 (69.2)	814 (63.6)	847 (62.2)	6,010 (64.8)
Divorced or separated	175 (13.1)	100 (8.0)	152 (11.3)	86 (6.4)	110 (8.2)	91 (7.1)	100 (7.3)	814 (8.8)
Widowed	131 (9.8)	96 (7.6)	83 (6.2)	92 (6.8)	142 (10.5)	109 (8.5)	113 (8.3)	766 (8.3)
Single	212 (15.8)	110 (8.8)	330 (24.5)	310 (23.0)	162 (12.0)	265 (20.7)	301 (22.1)	1,690 (18.2)
Religion or denomination								
With a religion or denomination	778 (57.9)	664 (52.9)	771 (57.0)	1,094 (81.6)	616 (45.6)	1,017 (79.6)	959 (71.0)	5,899 (63.6)

DE: Germany; EN: England; ES: Spain; FL: Flanders; IT: Italy; NL: Netherlands; PT: Portugal; SD: standard deviation.

^*^Sums may not always amount to the total sample number because of missing values on variables. Percentages may not always add up to 100 because of rounding.

^†^The percentage of missing data was 6.3% for least preferred place of death, 5.1% for age, 0.03% for gender, 0.6% for living arrangements, 0.7% for marital status, 1.1% for religion/denomination. Missing data include “don’t know”, refusals, interview break-offs and data missing from the computer-assisted telephone interviewing (CATI) system.

Overall mean age was 50.7 (standard deviation (SD) 16.0); 66.1% of participants were female and 92.7% were born in the country where the survey took place (Table 1). Ten percent reported having been seriously ill in the past five years and 53.1% had cared for a close relative or friend in their last months of life (see Additional file 4).

When participants were asked where they would least prefer to die if the circumstances allowed them to choose, care homes and hospitals were either the most or second most frequent answer in all countries. Care homes were the most common least preferred place in the Netherlands (41.5%), Italy and Spain (both 36.7%). In England (28.0%), Portugal (25.8%), Germany (23.7%) and Flanders (18.9%) they were the second most frequently chosen answer (the first was hospital).

### Care home as the least preferred place of death by participants’ characteristics

No variable was significantly associated with choosing care homes as the least preferred place of death across all countries. Age and religion/denomination were the only variables showing a consistent direction cross-nationally (Table 2). Older participants more often chose care homes as the least preferred place of death (p < 0.001), although within countries differences were only significant in Germany (p = 0.017) and the Netherlands (p = 0.021). Those belonging to a religion or denomination were more likely to see care homes as the least preferred place to die, but the results only reached statistical significance overall (p = 0.013). Other socio-demographics showed variable effects between countries (i.e. being unemployed, sick or disabled, doing housework, country of birth, and experiences of serious illness, death and dying).

**Table 2:** **Care homes as the least preferred place of death (n = 2,653) by participant’s characteristics (N = 9,344)**

	EN n(%)	FL n(%)	DE n(%)	IT n(%)	NL n(%)	PT n(%)	ES n(%)	All n(%)
Marital status		*						
Married or with a partner	229 (28.9)	168 (18.9)	176 (23.5)	285 (35.9)	381 (42.1)	193 (25.9)	275 (35.7)	1,707 (30.2)
Divorced or separated	38 (22.9)	27 (27.0)	29 (20.1)	27 (34.6)	44 (43.6)	17 (20.5)	31 (36.0)	213 (28.1)
Widowed	34 (28.1)	13 (14.9)	24 (31.2)	32 (39.0)	53 (39.8)	30 (32.3)	41 (45.1)	227 (33.2)
Single	60 (29.4)	13 (12.3)	78 (24.4)	111 (38.7)	62 (39.2)	63 (25.3)	102 (36.0)	489 (33.4)
Activities in the last seven days								
Unemployed				**				
Yes	22 (36.1)	12 (25.0)	9 (14.3)	23 (22.5)	17 (37.0)	27 (27.6)	57 (33.9)	167 (28.5)
No	340 (27.6)	212 (18.6)	298 (24.3)	434 (38.1)	523 (41.8)	276 (25.7)	395 (37.1)	2,478 (30.5)
Permanently sick or disabled		**						
Yes	14 (21.9)	15 (35.7)	41 (26.8)	2 (22.2)	39 (37.5)	12 (34.3)	17 (37.0)	140 (30.9)
No	348 (28.4)	209 (18.2)	266 (23.4)	455 (36.9)	501 (42.0)	291 (25.5)	435 (36.7)	2,505 (30.3)
Doing housework			**		*			
Yes	114 (29.9)	62 (20.5)	87 (19.2)	112 (36.1)	130 (36.2)	49 (24.9)	121 (36.1)	675 (28.9)
No	248 (27.3)	162 (18.2)	220 (26.3)	345 (37.0)	410 (43.7)	254 (26.0)	331 (36.9)	1,970 (30.9)
Age								
Mean age	54.3 vs 53.9	52.3 vs 51.9	48.9 vs 46.4	48.9 vs 47.4	55.5 vs 53.4	50.4 vs 48.9	47.8 vs 47.0	51.2 vs 49.9
Age bands		*	*		*			**
16-29	30 (28.8)	9 (10.5)	40 (19.8)	65 (38.2)	19 (31.7)	40 (24.8)	71 (36.6)	274 (28.0)
30-39	39 (26.9)	22 (20.0)	33 (17.6)	48 (30.8)	43 (34.1)	42 (24.9)	72 (35.6)	299 (27.2)
40-49	62 (24.8)	59 (23.8)	89 (25.5)	67 (30.3)	109 (38.9)	57 (25.7)	95 (36.5)	538 (29.4)
50-59	75 (30.0)	63 (21.2)	71 (27.2)	107 (42.8)	145 (47.7)	52 (23.6)	92 (36.1)	605 (32.9)
60-69	88 (28.8)	42 (17.4)	43 (24.2)	75 (42.9)	121 (41.4)	57 (27.9)	70 (40.9)	496 (31.6)
70+	63 (28.4)	23 (20.2)	31 (28.7)	34 (34.0)	82 (44.1)	43 (30.1)	46 (40.0)	322 (32.6)
Born in country			*		*			**
Yes	326 (28.3)	218 (19.2)	290 (24.6)	435 (36.5)	518 (42.2)	280 (26.4)	419 (36.5)	2,486 (30.7)
No	36 (25.5)	8 (13.3)	18 (15.0)	22 (43.1)	23 (30.3)	24 (20.7)	35 (41.2)	166 (25.6)
Gender			*					
Male	129 (27.9)	83 (20.3)	147 (27.2)	124 (35.9)	177 (40.0)	82 (23.3)	128 (33.0)	870 (29.6)
Female	232 (28.0)	143 (18.2)	161 (21.2)	333 (37.0)	365 (42.4)	222 (26.9)	326 (38.4)	1,782 (30.7)
Experience caring for close relative/friend in last months of life				*				**
Yes	186 (28.4)	119 (20.1)	151 (24.3)	292 (39.3)	293 (42.8)	167 (26.6)	250 (36.1)	1,458 (31.6)
No	175 (27.6)	105 (17.8)	153 (23.0)	160 (32.5)	248 (40.2)	135 (25.2)	199 (37.3)	1,175 (28.9)
Close relative/friend with serious illness								*
Yes	243 (29.7)	142 (19.7)	197 (23.8)	302 (36.1)	397 (42.3)	166 (24.3)	313 (37.5)	1,760 (31.1)
No	119 (25.3)	80 (17.3)	106 (23.1)	149 (38.0)	144 (39.7)	136 (28.4)	137 (34.9)	871 (28.9)
Religion or denomination								*
Yes	216 (29.1)	121 (19.6)	178 (24.1)	374 (37.5)	255 (43.2)	249 (27.1)	317 (36.9)	1,710 (31.3)
No	143 (26.3)	105 (18.5)	129 (23.3)	80 (33.9)	285 (40.1)	55 (21.8)	131 (36.0)	928 (28.7)

DE: Germany; EN: England; ES: Spain; FL: Flanders; IT: Italy; NL: Netherlands; PT: Portugal; Vs: versus.

Only variables found to be significant in bivariate analysis for at least one country or all countries together are shown here. *P values ≤0.05. **P values ≤0.01.

### Country models and factors associated with choosing care homes as the least preferred place

We found three factors independently associated with choosing care homes as the least preferred place to die in more than one country (Table 3). Participants doing housework in the past seven days were less likely to see care homes as the least preferred place to die in Germany (AOR 0.72; 95% CI:0.54-0.96) and in the Netherlands (AOR 0.68; 95% CI:0.52-0.90). Also in Germany and the Netherlands, those who were born in the country where the survey took place were more likely to choose care homes as the least preferred place of death (AOR 1.77; 95% CI 1.05-2.99 and AOR 1.74; 95% CI 1.03-2.95 respectively). Those for whom keeping a positive attitude was a top end of life priority were more likely to see care homes as the least preferred place to die in Italy (AOR 1.48; 95% CI:1.04-2.11), whilst in Portugal it was the other way around (AOR 0.69; 95% CI:0.51-0.93).

**Table 3:** **Factors associated with care home as the least preferred place (in bold) and model fitting statistics**

	EN	FL	DE	IT	NL	PT	ES
	AOR (95% CI)	AOR (95% CI)	AOR (95% CI)	AOR (95% CI)	AOR (95% CI)	AOR (95% CI)	AOR (95% CI)
**Variables in the model**							
Age (*ref 16–49*)							
50+	1.12 (0.87–1.45)	0.96 (0.69–1.33)	1.24 (0.95–1.62)	1.20 (0.89–1.61)	**1.40 (1.10–1.79)**	0.97 (0.73–1.29)	1.11 (0.86–1.42)
Born in country*(ref born overseas)*	-	-	**1.77 (1.05–2.99)**	-	**1.74 (1.03–2.95)**	-	-
Unemployed in last seven days *(ref no)*	-	-	-	**0.47 (0.27–0.83)**	-	-	-
Doing housework in last seven days *(ref no)*	-	-	**0.72 (0.54–0.96)**	-	**0.68 (0.52–0.90)**	-	-
Permanently sick or disabled in last seven days *(ref no)*	-	**2.34 (1.17–4.69)**	-	-	-	-	-
Wanting information about symptoms and problems (ref Yes, but only if ask for it/No)							
Yes, always	-	-	-	**0.64 (0.45–0.89)**	-	-	-
Concerns about symptoms and problems							
Being in pain as top concern (*ref no*)	-	**1.56 (1.06–2.30)**	-	-	-	-	-
Being alone as top concern (*ref no*)	-	-	-	1.31 (0.96–1.81)	-	-	**1.41 (1.07–1.86)**
Decision making in capacity and incapacity scenarios							
Partner deciding in a scenario of capacity *(ref no)*	-	-	-	**1.78 (1.25–2.53)**	-	-	-
Doctor deciding in a scenario of capacity *(ref no)*	-	-	-	-	-	**1.45 (1.08–1.94)**	-
Oneself deciding in a scenario of incapacity *(ref no)*	-						**1.44 (1.12–1.84)**
Other relative deciding in a scenario of incapacity *(ref no)*	-	-	-	**2.09 (1.48–2.96)**	-	-	-
Keeping positive attitude top priority (ref no)	-	0.83 (0.60–1.15)	-	**1.48 (1.04–2.11)**	-	**0.69 (0.51–0.93)**	-
Quality of life versus life extension (ref extending life most important)							
Improving quality most important priority	-	-	-	-	1.58 (0.86–2.92)	-	-
Both equally important	-	-	-	-	**2.18 (1.14–4.17)**	-	-
**Model-fitting statistics**							
Nagelkerke R2	0.002	0.040	0.033	0.119	0.029	0.021	0.017
Hosmer and Lemeshow test - χ2(df) = chi-square, p-value	χ2(2) = 0.056, p = 0.973	χ2(8) = 15.554, p = 0.049	χ2(8) = 6.007, p = 0.646	χ2(8) = 7.668, p = 0.467	χ2(8) = 8.287, p = 0.406	χ2(8) = 1.382, p = 0.995	χ2(7) = 4.247, p = 0.751
Area under the ROC curve	0.521	0.603	0.601	0.673	0.581	0.574	0.569
Care homes classified correctly as least preferred place,%	0.0%	1.4%	0.0%	29.5%	9.3%	0.0%	1.0%

CI: confidence intervals; DE: Germany; df: degrees of freedom; EN: England; ES: Spain; FL: Flanders; IT: Italy; NL: Netherlands; OR: odds ratio; PT: Portugal; ROC: receiver operating characteristic.

- Variable not in regression model in this country. Significant variables in bold. Table excludes variables which were included in the models for being significant in the bivariate analysis but were not significant in any country after adjusting for confounders. Valid cases per model: 1271 in England, 1040 in Flanders, 1261 in Germany, 910 in Italy, 1219 in the Netherlands, 1065 in Portugal and 1127 in Spain.

Different factors were associated with the preference in different countries. In Flanders, those who chose being in pain as a top concern were more likely to choose care homes as their least preferred place (AOR 1.56; 95% CI: 1.06-2.30). In Spain, those who would like to make decisions in a scenario of incapacity (AOR 1.44; 95% CI: 1.12-1.84) or who chose being alone as a top concern (AOR 1.41; 95% CI: 1.07-1.86) were also more likely to choose care homes as their least preferred place. England was the only country with no significant factors associated with the preference while Italy had the highest number of associated factors (five).

There was no country in which the association of previous experience of serious illness, death and dying with choosing care homes was significant, except that participants who were permanently sick or disabled in Flanders were more likely to choose care homes as their least preferred place (AOR 2.34; 95% CI: 1.17-4.69).

Country models explained a small part of the variance in each in individual country; Nagelkerke R2 (%) ranged from 0.2% in England to 11.9% in Italy. The models were unable to differentiate well people who saw care homes as the least preferred to die from others (the highest percentage correctly classified was 29.5% for Italy).

## Discussion

To our knowledge, this is the first study to examine the general population’s least preferred place of death across Europe, focusing on factors associated with care homes as the least preferred place. We found that, despite variations across countries, care homes were either the first or the second (most common) least preferred place to die. By using a robust research methodology and questions comparable across countries, we have generated country-specific and cross-national evidence on public preferences to inform policymaking in Europe. Public views have been found to generate good quality, clinically relevant research [31–33]. Furthermore, the public has the right to be involved in research which may have an impact on their health or decisions which will be made about their care in the future [31, 32, 34].

Some study limitations should be highlighted. Since we have focused our analysis on care homes we did not discuss negative preferences regarding other settings, but it is important to note that hospitals were also frequently chosen as the least preferred place of death. Additionally, our survey questions were phrased to allow for different experiences of advanced illness, but suggested a scenario of cancer. Hence, we could not investigate preferences according to different conditions, and bias towards a scenario of advanced cancer is possible. Furthermore, in order to compare preferences across countries we merged ‘nursing’ and ‘residential homes’ in England and the Netherlands. These facilities involve different levels of nursing care and are particularly different in the Netherlands, hence people’s views on them might differ [35, 36]. Our response rate was low, a typical problem when using RDD [37]. We also had an over-representation of women and older people. However, older people and women are more likely to be care home residents and listening to their views is especially relevant [38, 39]. Survey participants were mostly healthy, and evidence suggests that preferences for institutionalised care can vary according to the level of need and disability [8]. We found no influence of experience of serious illness, death and dying (questions 24–27 in the questionnaire), although in Belgium those permanently sick or disabled (question 22) were more likely to choose care homes as the least preferred place to die. These participants may have greater concerns regarding losing control and ability to care for themselves, and this might be negatively associated with moving to a care home to spend the final stages of their lives [40]. Nevertheless, such difference was found in only one country.

Sixty-five percent of our sample chose either hospital or care home as their least preferred place. In contrast, hospices and palliative care units (the other institutional settings provided as an answer option) were chosen as the least preferred place by only 8.5% of the participants (lower than the percentage for home). Perhaps this is due to the availability of palliative and patient-centred care in these settings. Evidence shows that hospice is often the second most frequently chosen place to die (after home) [24, 41].

In England, where care homes were chosen as the least preferred place by 28.0% of participants, no factors were found to be associated with this preference. Previous analyses from this survey have shown that home was chosen as the most preferred place of death by 63.0% of participants in this country (the percentage for care home was 2.0%) [24]. Our results suggest that people do not see these care settings as equivalent places of death, although they are part of the same quality marker in end of life care provision (death in the usual place of residence). Since meeting people’s preferences at the end of life is a priority for the National Health Services in England [11], it is crucial that people’s views regarding care homes are further investigated.

There was not a single factor consistently associated with the preference across Portugal, Italy and Spain, which are commonly grouped together as “Southern European Countries” in studies about culture and end of life care [42]. Perhaps this is not so surprising considering remarkable cross-national differences in terms of service provision and palliative care availability [18]. The only factors associated with the preference in more than one country had opposite directions (keeping a positive attitude in Portugal and Italy). This unexpected contradiction is difficult to interpret in the absence of further evidence.

Results from Portugal are different from the other countries regarding the proportion of participants choosing own home as the least preferred place to die (21.8%, the highest percentage across all countries). This may be due to the limited availability of palliative care services for patients who remain at home [43]. The negative preference towards care homes may be associated with availability and affordability of these services. In this country there is a strong reliance in informal and privately funded care [44]. Hence, care homes might not be seen as a possible option.

Italy was the country with the highest number of factors associated with the preference. Italians often prefer to die in a calm atmosphere surrounded by close relatives and friends [42]. Care homes may be associated with being apart from family, which has a fundamental role in informal caregiving in this country [45]. Those who always wanted information about their symptoms and problems were less likely to choose care homes. Family caregivers have also been reported as a barrier to full disclosure of information [42]; this specific group of participants might have had fewer reservations towards a care home as in this setting they could potentially have fewer barriers to information.

In Spain those who were concerned about being alone were more likely to choose care homes as their least preferred place; so were those who would like to make their own decisions about care in a scenario of incapacity. In this country advanced directives are well-developed [45]. These respondents might have felt that their registered wishes would not be respected in a care home if they had lost their autonomy. The fear about being alone and the negative preference towards care homes might be attributed to the importance of being with family. Other studies have shown that in Spain over 80% of patients are cared for by family members; this would not be possible in a care home [45].

The case of the Netherlands and Germany deserves special attention. Despite good care home availability [39] and the highest proportion of care home deaths in Europe [46], the Netherlands had the highest percentage of people choosing care homes as their least preferred place. Care homes have become highly medicalised in this country, resembling hospital care with large wards and bedrooms for multiple residents [47]. Evidence from the Netherlands also shows that care homes could benefit from more patient-centred care and increased palliative care availability, in addition to a stronger focus on symptom control [4, 39, 48]. In this country, we saw that people who said improving quality of life and extending life were both equally important priorities were more likely to see care homes as the least preferred place to die. This group might have felt that living and dying in care homes was not compatible with their priorities. The Netherlands was also the only country to show a significant association of age and choosing care homes as the least preferred place after adjusting for confounders. Older people were more likely to choose care home as their least preferred place compared with their younger counterparts. Similar to the preferences from participants who are permanently sick or disabled, it is possible that older participants saw care home as a sign of loss of control and autonomy (especially considering that when the survey was carried out they were still living at home). Having to make choices about place of death might also have been much less hypothetic for them than for the younger age group. It is not clear, however, why age was only significant in the Netherlands (as the same explanations could be applied to any of the investigated countries).

Both in Germany and the Netherlands those doing housework were less likely to choose care homes as the least preferred place to die, regardless of gender. Since they are doing the household chores (35.3% of respondents in Germany and 27.6% in the Netherlands), they might have concerns on whether there would be someone else available to help them in case they needed care at home. In these cases care homes might be seen as an alternative option.

Our multivariate binary logistic regression models were not able to explain much variance in each country. Personal knowledge and having relatives living in care homes are possible influencing factors that we did not cover. A recent study with the general public in Germany found that although care homes often had a poor image, views on some aspects of care were more positive from those who had concrete knowledge about these settings. Nonetheless, issues such as poor provision of personal care and the need for more staff training were common concerns [49]. The need to improve care and symptom control in care homes [4, 39, 50, 51] and their poor public image, in addition to the fact that they are many times inadequately resourced [4, 13–15] could also play a role. The fact that care homes have become a place where people go to die [6] might also contribute to a negative perception of this setting. Care homes might be associated with people living with dementia and high dependency to others [52].

## Conclusions

This study shows that care homes are seen as the least preferred place to die by a substantial proportion of study participants in all the investigated countries, with cross-national variations on factors underlying this negative preference. Our results suggest that dying at home and in a care home is not viewed as similar and that it might be challenging to promote care homes as a good place to die. Furthermore, since a substantial number of people who die in care homes are likely to have lived in this setting before death, promoting care homes as places of care for older people reaching the end of life may also be difficult. This possibility needs to be further explored in studies investigating the general population’s views regarding both living and dying in care homes.

Further research should investigate the views of care home residents; this group might see care homes as their homes [52] and have a different perspective from the general population. Reviews analysing the portrayal of nursing homes in the media may be helpful to assess whether negative reports can affect public perceptions of this setting [53]. Qualitative studies are suggested to explore in depth the reasons why people do not wish to die in care homes, including possible concerns regarding dependency on others [8], loss of autonomy and personal control [52, 54] in these settings. Learning from the experiences of inpatient hospices, which are seen favourably by patients, might also be a way forward. This is an urgent area of research in order to meet needs and preferences of a growing number of older people with chronic, debilitating conditions across Europe.

## Electronic supplementary material

Additional file 1: **Survey questionnaire (English only).** (DOC 100 KB)

Additional file 2: **Questions on preferences for place of death.** (DOC 51 KB)

Additional file 3: **Variables tested in bivariate analysis.** (DOC 35 KB)

Additional file 4: **Additional participant demographics and experiences of illness, death and dying by country.** (DOCX 21 KB)

## References

[1] Gomes B, Cohen J, Deliens L, Higginson IJ, Gott M, Ingleton C (2011). International trends in circumstances of death and dying. Living with Ageing and Dying Palliative and End of Life Care for older People.

[2] Calanzani N, Higginson IJ, Gomes B (2013). Current and Future Needs for Hospice Care: an Evidence-based Report.

[3] Damiani G, Farelli V, Anselmi A, Sicuro L, Solipaca A, Burgio A, Iezzi DF, Ricciardi W (2011). Patterns of Long Term Care in 29 European countries: evidence from an exploratory study. BMC Health Serv Res.

[4] van der Steen J, Helton MR, Sloane PD, Ribbe MW, Cohen J, Deliens L (2012). Palliative care in institutional long-term care settings. A Public Health Perspective on End of Life Care.

[5] Office for National Statistics: **Mortality Statistics. Deaths Registered in England and Wales 2012. Table 13. Deaths: area of usual residence and sex, by place of occurrence, numbers and percentages.** http://www.ons.gov.uk/ons/publications/re-reference-tables.html?edition=tcm%3A77-325289

[6] Houttekier D, Cohen J, Bilsen J, Addington-Hall J, Onwuteaka-Philipsen BD, Deliens L (2010). Place of death of older persons with dementia. A study in five European countries. J Am Geriatr Soc.

[7] Gomes B, Calanzani N, Gysels M, Hall S, Higginson IJ (2013). Heterogeneity and changes in preferences for dying at home: a systematic review. BMC Palliat Care.

[8] Wielink G (1997). Elderly Community Resident’s Preferences for Care. A Study of Choices and Determinants in Hypothetical Care-Need Situations. PhD Thesis.

[9] World Health Organization (2011). Palliative care for Older People: Better Practices.

[10] Beccaro M, Costantini M, Giorgi Rossi P, Miccinesi G, Grimaldi M, Bruzzi P (2006). Actual and preferred place of death of cancer patients. Results from the Italian survey of the dying of cancer (ISDOC). J Epidemiol Community Health.

[11] Department of Health (2012). End of Life Care Strategy: Fourth Annual Report.

[12] Eurostat: **Mortality and life expectancy statistics.** http://epp.eurostat.ec.europa.eu/statistics_explained/index.php/Mortality_and_life_expectancy_statistics

[13] Santana S, Dias A, Souza E, Rocha N (2007). The Domiciliary Support Service in Portugal and the change of paradigm in care provision. Int J Integr Care.

[14] OECD: **Health at a Glance 2011: OECD Indicators.** http://www.oecd.org/els/healthpoliciesanddata/49105858.pdf

[15] Knapp M, Comas-Herrera A, Somani A, Banerjee S: **Dementia: international comparisons. PSSRU Discussion Paper 2418.** http://www.pssru.ac.uk/pdf/dp2418.pdf

[16] Cohen J, Houttekier D, Onwuteaka-Philipsen B, Miccinesi G, Addington-Hall J, Kaasa S, Bilsen J, Deliens L (2010). Which patients with cancer die at home? A study of six European countries using death certificate data. J Clin Oncol.

[17] Gomes B, Higginson IJ (2008). Where people die (1974–2030): past trends, future projections and implications for care. Palliat Med.

[18] Centeno C, Lynch T, Donea O, Rocafort J, Clark D (2013). EAPC Atlas of Palliative Care in Europe 2013.

[19] Froggatt K, Reitinger E, Heimerl K, Hockley J, Brazil K, Kuntz R, Parker D, Sandgathe-Husebo B, Morbey H (2013). Palliative Care in Long-Term Care Settings for Older People. EAPC Taskforce 2010–2012.

[20] ANCIEN: **Assessing Needs of Care In European Nations.** http://www.ancien-longtermcare.eu/

[21] Harding R, Higginson IJ, on behalf of PRISMA (2010). A pan-European co-ordinating action to advance the science in end-of-life cancer care. Eur J Cancer.

[22] Triandis H (1977). Interpersonal Behavior.

[23] Bronfenbrenner U (1979). The Ecology of Human Development: Experiments by Nature and Design.

[24] Gomes B, Higginson I, Calanzani N, Cohen J, Deliens L, Daveson BA, Bechinger-English D, Bausewein C, Ferreira PL, Toscani F, Meñaca A, Gysels M, Ceulemans L, Simon S, Pasman H, Albers G, Hall S, Murtagh FE, Haugen D, Downing J, Koffman J, Pettenati F, Finetti S, Antunes B, Harding R, on behalf of PRISMA (2012). Preferences for place of death if faced with advanced cancer: a population survey in England, Flanders, Italy, Germany, the Netherlands, Portugal and Spain. Ann Oncol.

[25] Daveson BA, Bechinger-English D, Bausewein C, Simon S, Harding R, Higginson IJ, Gomes B (2011). Constructing understandings of end-of-life care in Europe: a qualitative study involving cognitive interviewing with implications for cross-national surveys. J Palliat Med.

[26] Bausewein C, Calanzani N, Daveson BA, Simon ST, Ferreira PL, Higginson IJ, Bechinger-English D, Deliens L, Gysels M, Toscani F, Ceulemans L, Harding R, Gomes B, Prisma (2013). ‘Burden to others’ as a public concern in advanced cancer: a comparative survey in seven European countries. BMC Cancer.

[27] Daveson BA, Bausewein C, Murtagh FE, Calanzani N, Higginson IJ, Harding R, Cohen J, Simon ST, Deliens L, Bechinger-English D, Hall S, Koffman J, Ferreira PL, Toscani F, Gysels M, Ceulemans L, Haugen DF, Gomes B, Prisma (2013). To be involved or not to be involved: a survey of public preferences for self-involvement in decision-making involving mental capacity (competency) within Europe. Palliat Med.

[28] Harding R, Simms V, Calanzani N, Higginson IJ, Hall S, Gysels M, Menaca A, Bausewein C, Deliens L, Ferreira P, Toscani F, Daveson BA, Ceulemans L, Gomes B, on behalf of PRISMA (2013). If you had less than a year to live, would you want to know? A seven-country European population survey of public preferences for disclosure of poor prognosis. Psychooncology.

[29] Higginson IJ, Gomes B, Calanzani N, Gao W, Bausewein C, Daveson BA, Deliens L, Ferreira PL, Toscani F, Gysels M, Ceulemans L, Simon ST, Cohen J, Harding R, on behalf of Project Prisma (2014). Priorities for treatment, care and information if faced with serious illness: a comparative population-based survey in seven European countries. Palliat Med.

[30] Daveson BA, Alonso JP, Calanzani N, Ramsenthaler C, Gysels M, Antunes B, Moens K, Groeneveld EI, Albers G, Finetti S, Pettentati F, Bausewein C, Higginson IJ, Harding R, Deliens L, Toscani F, Ferreira PL, Ceulemans L, Gomes B, on behalf of Prisma (2014). Learning from the public: citizens describe the need to improve end-of-life care access, provision and recognition across Europe. Eur J Public Health.

[31] Boote J, Baird W, Beecroft C (2010). Public involvement at the design stage of primary health research: a narrative review of case examples. Health Policy.

[32] Boote J, Telford R, Cooper C (2002). Consumer involvement in health research: a review and research agenda. Health Policy.

[33] Entwistle V, Renfrew M, Yearley S, Forrester J, Tara J (1998). Lay perspectives: advantages for health research. BMJ.

[34] Barber R, Boote JD, Parry GD, Cooper CL, Yeeles P, Cook S (2012). Can the impact of public involvement on research be evaluated? A mixed methods study. Health Expect.

[35] Abarshi E (2011). Care in the last months of life. End-of-Life Care registration in the Netherlands by a Network of General Practitioners. PhD Thesis.

[36] Abarshi E, Echteld MA, Van den Block L, Donker G, Deliens L, Onwuteaka-Philipsen B (2010). The oldest old and GP end-of-life care in the Dutch community: a nationwide study. Age Ageing.

[37] Kempf AM, Remington PL (2007). New challenges for telephone survey research in the twenty-first century. Annu Rev Public Health.

[38] Ruth K, Verne J: **Deaths in Older Adults in England.** http://www.endoflifecare-intelligence.org.uk/view.aspx?rid=82

[39] Abarshi E, Echteld MA, Van den Block L, Donker G, Bossuyt N, Meeussen K, Bilsen J, Onwuteaka-Philipsen B, Deliens L (2011). Use of palliative care services and general practitioner visits at the end of life in the Netherlands and Belgium. J Pain Symptom Manage.

[40] Van Garderen F, Vandekerckhove S (2014). Angst voor het rusthuis. De Morgen.

[41] Higginson IJ, Sen-Gupta GJ (2000). Place of care in advanced cancer: a qualitative systematic literature review of patient preferences. J Palliat Med.

[42] Meñaca A, Evans N, Andrew E, Toscani F, Finetti S, Gómez-Batiste X, Higginson I, Harding R, Pool R, Gysels M (2012). End-of-life care across Southern Europe: a critical review of cultural similarities and differences between Italy, Spain and Portugal. Crit Rev Oncol Hematol.

[43] Gomes B, Sarmento VP, Ferreira PL, Higginson IJ (2013). Epidemiological study of place of death in Portugal in 2010 and comparison with the preferences of the Portuguese population. Acta Med Port.

[44] Santana S (2010). Reforming long-term care in Portugal: dealing with the multidimensional character of quality. Soc Policy Admin.

[45] Gysels M, Evans N, Menaca A, Andrew E, Toscani F, Finetti S, Pasman HR, Higginson I, Harding R, Pool R (2012). Culture and end of life care: a scoping exercise in seven European countries. PLoS One.

[46] Andrew E, Cohen J, Evans N, Meñaca A, Harding R, Higginson IJ, Pool R, Gysels M, PRISMA (2013). Social-cultural factors in end-of-life care in Belgium: a scoping of the research literature. Palliat Med.

[47] Boekhorst S, Pot A, Depla M, Smit D, Lange J, Eefsting J (2008). Group living homes for older people with dementia: the effects on psychological distress of informal caregivers. Aging Ment Health.

[48] Brandt HE, Deliens L, van der Steen JT, Ooms ME, Ribbe MW, van der Wal G (2005). The last days of life of nursing home patients with and without dementia assessed with the palliative care outcome scale. Palliat Med.

[49] Institut für Demoskopie Allensbach (2009). Pflege in Deutschland. Ansichten der Bevölkerung über Pflegequalität und Pflegesituation.

[50] Carlson AL (2007). Death in the nursing home: resident, family, and staff perspectives. J Gerontol Nurs.

[51] Pinzón LCE, Claus M, Zepf KI, Fischbeck S, Weber M (2012). Symptom prevalence in the last days of life in Germany: the role of place of death. Am J Hosp Palliat Med.

[52] Froggatt K (2005). ‘Choice over care at the end of life’: implications of the end of life care initiative for older people in care homes. J Res Nurs.

[53] Miller E, Tyler D, Mor V (2013). National newspaper portayal of nursing homes: tone of coverage and its correlates. Med Care.

[54] Scott PA, Välimäki M, Leino-Kilpi H, Dassen T, Gasull M, Lemonidou C, Arndt M, Schopp A, Suhonen R, Kaljonen A (2003). Perceptions of autonomy in the care of elderly people in five European countries. Nurs Ethics.

[CR55] The pre-publication history for this paper can be accessed here:http://www.biomedcentral.com/1472-684X/13/48/prepub

