# Supplementary material for: Choosing care homes as the least preferred place to die: a cross-national survey of public preferences in seven European countries
Source: BMC Palliat Care. 2014 Oct 23;13:48. doi: 10.1186/1472-684X-13-48 (PMC4430987; doi:10.1186/1472-684X-13-48)
Supplement: Supplementary file 1 — Additional file 1: Survey questionnaire (English only). (DOC 100 KB) [file 12904_2014_229_MOESM1_ESM.doc]

**Additional file 1: Survey questionnaire (English only)**

1. PRISMA questions

I will now ask you to imagine a situation of serious illness, for example cancer, with less than one year to live. Please keep this in mind for the rest of the interview, continue to imagine this situation as you answer to the questions.

Q1. If you had a serious illness, for example cancer, and were likely to have less than one year to live, would you like to be informed that you had limited time left?

1 🞎 Yes, always

2 🞎 Yes, but only if you ask about it

3 🞎 No

97 🞎 You don’t know

Q2. Would you like to be informed about what symptoms and problems you were likely to experience?

1 🞎 Yes, always

2 🞎 Yes, but only if you ask about it

3 🞎 No

97 🞎 You don’t know

Q3. Would you like to be informed about the options available for care and how they might affect you? These options might be services available, places where you could be looked after, treatments and medication.

1 🞎 Yes, always

2 🞎 Yes, but only if you ask about it

3 🞎 No

97 🞎 You don’t know

Q4. Which of the following nine symptoms or problems do you think would concern you the most? I will now read out the nine symptoms or problems. If you have a pen and paper at hand, it may help to write these down. Otherwise, I can read the list as many times as you need.

|  | A | B |
| --- | --- | --- |
| **List of problems** | 1^st^ place  (1^st^ most concerning) | 2^nd^ place  (2^nd^ most concerning) |
| 1. Having no energy | 1 | 2 |
| 2. Being in pain | 1 | 2 |
| 3. Changes in the way you look | 1 | 2 |
| 4. Having no appetite at all | 1 | 2 |
| 5. Being a burden to others | 1 | 2 |
| 6. Being unable to get your breath | 1 | 2 |
| 7. Being alone | 1 | 2 |
| 8. Feeling as if you want to be sick | 1 | 2 |
| 9. Being worried and distressed | 1 | 2 |

1. So which one of these do you think would concern you most?
2. And in **second place**? I can read the list again if you would like me to.

Q5. Keeping in mind a situation of serious illness with less than one year to live, please consider that you were able to make decisions. Who would you like to make decisions about your care? **Please choose as many as apply**, you can choose more than one.

1 🞎 Yourself

2 🞎 Your spouse or partner

3 🞎 Other relatives

4 🞎 Friends

5 🞎 The doctor

95 🞎 Others _______________________

Q6. What if you had lost your ability to make decisions, who would you like to make decisions about your care? **Please choose as many as apply**, you can choose more than one.

1 🞎 Yourself, by specifying your wishes before losing ability - for example, in a living will

2 🞎 Your spouse or partner

3 🞎 Other relatives

4 🞎 Friends

5 🞎 The doctor

95 🞎 Others

Q7. In a situation of serious illness like cancer with less than one year to live....

|  | A | B |
| --- | --- | --- |
| **Answer options** | Most preferred | Least preferred |
| In your own home | 1 | 1 |
| In the home of a relative or friend | 2 | 2 |
| In a hospice or palliative care unit - places with specialised care and beds for dying patients | 3 | 3 |
| In hospital - but not in a palliative care unit | 4 | 4 |
| In a nursing home | 5 | 5 |
| In a residential home | 6 | 6 |
| Somewhere else: _______________ | 95 | 95 |

1. Where do you think you would prefer to die **if circumstances allowed you to choose**? I will now read out the answer options.
2. And which of these do you think you would least prefer if circumstances allowed **you to choose**?

Q8. When people are faced with a serious illness like cancer with limited time to live, they may have to make difficult decisions and prioritise some things over others. In this situation, how would you order the following four aspects by their level of importance to you, the first being the most important (1) and the last being the least important (4)?

I will now read you the four things. Take your time to think about each thing and to order them as you think best describes how you would feel in that situation. I can read them again if you would like.

| **Aspects** | RANK  1= MOST IMPORTANT/ 4= LEAST IMPORTANT |
| --- | --- |
| A - Keeping a positive attitude |  |
| B - Having pain and discomfort relieved |  |
| C - Having practical matters resolved |  |
| D - Making sure relatives and friends are not worried or distressed |  |

Q9. What would matter most to you in the care available? Please choose from the following three aspects the one that would matter most to you.

|  | 1^st^ most important | 2^nd^ most important |
| --- | --- | --- |
| 1 Having as much information as you want | 1 | 2 |
| 2 Choosing who makes decisions about your care | 1 | 2 |
| 3 Dying in the place you want | 1 | 2 |

1. Please choose the one you think would matter most to you.
2. And in **second place**? I can read them again if you would like me to.

Q10. When people are faced with a serious illness like cancer with limited time to live, they may have to make difficult decisions and prioritise some things over others. In this situation, would it be more important to extend your life or to improve the quality of life for the time you had left?

1 🞎 To extend life

2 🞎 To improve the quality of life for the time you had left

3 🞎 Both are **equally** important

97 🞎 You don’t know

**2. Socio-demographic questions**

Q11. How old are you?

| Age |  | Write down the age in years |
| --- | --- | --- |

Q12. Would you describe yourself as male or female?

| 1 🞎 Male |
| --- |
| 2 🞎 Female |

Q13. In which country were you born?

1 🞎 England

2 🞎 Other _______________

Q14. How long ago did you first come to live in England?

| 1 🞎 Within last year |
| --- |
| 2 🞎1-5 years ago |
| 3 🞎 6-10 years ago |
| 4 🞎 11-20 years ago |
| 5 🞎More than 20 years ago |

Q15. Which best describes the area where you live?

| 1 🞎 A big city |
| --- |
| 2 🞎 Suburbs or outskirts of big city |
| 3 🞎 Town or small city |
| 4 🞎 Country village |
| 5 🞎 Farm or home in countryside |

# Q16. What is the highest level of education you have achieved?

| 1 🞎 No qualifications |
| --- |
| 2 🞎 GCSE/O-level/CSE/NVQ1/NVQ2 or equiv |
| 3 🞎 A-level/NVQ3 or equiv |
| 4 🞎 NVQ4/NVQ5 or equiv |
| 5 🞎 Degree/HNC/teacher training/nursing or equiv |
| 6 🞎 PhD/DPhil or equiv |
| 95 🞎 Other......................... |

Q17A. Do you consider yourself as belonging to any particular religion or denomination?

| 1 🞎 Yes |
| --- |
| 2 🞎 No |

Q17B. If yes, which one?

| 1 🞎 Roman Catholic |
| --- |
| 2 🞎 Church of England / Anglican |
| 3 🞎 Church or Ireland |
| 4 🞎 Baptist |
| 5 🞎 Methodist |
| 6 🞎 Presbyterian / Church of Scotland |
| 7 🞎 United Reform Church/Congregational |
| 8 🞎 Free Presbyterian |
| 9 🞎 Brethren |
| 10 🞎 Other Protestant (WRITE IN) ___________________ |
| 11 🞎 Greek or Russian Orthodox |
| 12 🞎 Other Eastern Orthodox (WRITE IN) _____________ |
| 13 🞎 Other Christian (WRITE IN) ____________________ |
| 14 🞎 Hindu |
| 15 🞎 Sikh |
| 16 🞎 Buddhist |
| 17 🞎 Other Eastern Religions (WRITE IN) _____________ |
| 18 🞎 Jewish |
| 19 🞎 Islam / Muslim |
| 20 🞎 Other non-Christian (WRITE IN) _________________ |
| 95 🞎 Other ____________________ |

Q18. How is your health (physical and mental) in general? Would you say it is:

| 1 🞎 Very good |
| --- |
| 2 🞎 Good |
| 3 🞎 Fair |
| 4 🞎 Bad |
| 5 🞎 Very bad |

Q19. Could I ask about your current legal marital status? Which of the descriptions apply to you?

| 1 🞎 Married |
| --- |
| 2 🞎 In a civil partnership |
| 3 🞎 Separated (still legally married) |
| 4 🞎 Separated (still in a civil partnership) |
| 5 🞎 Divorced |
| 6 🞎 Widowed |
| 7 🞎 Formerly in civil partnership, now dissolved |
| 8 🞎 Formerly in civil partnership, partner died |
| 9 🞎 Never married and never in civil partnership (single) |

Q20. How many adults, including yourself, aged 16 and over live in this household?

| 1 🞎 One (myself) |
| --- |
| 2 🞎 Two |
| 3 🞎 Three |
| 4 🞎 Four |
| 5 🞎 Five |
| 6 🞎 Six or more |

Q21. How many children aged under 16 live in this household?

| 1 🞎 None |
| --- |
| 2 🞎 One |
| 3 🞎 Two |
| 4 🞎 Three |
| 5 🞎 Four |
| 6 🞎 Five |
| 7 🞎 Six or more |

Q22. Which of the following descriptions applies to what you have been doing for the last 7 days? Select all that apply.

| 1 🞎 In paid work (or away temporarily) (employee, self-employed, working for your family business) |
| --- |
| 2 🞎 In education (not paid for by employer) even if on vacation |
| 3 🞎 Unemployed and actively looking for a job |
| 4 🞎 Unemployed, wanting a job but not actually looking for a job |
| 5 🞎 Permanently sick or disabled |
| 6 🞎 Retired |
| 7 🞎 In community or military service |
| 8 🞎 Doing housework, looking after children or other persons |
| 9 🞎 Other |

Q23. Which of the following descriptions comes closest to how you feel about your household’s income nowadays?

| 1 🞎 Living comfortably on present income |
| --- |
| 2 🞎 Coping on present income |
| 3 🞎 Difficult on present income |
| 4 🞎 Very difficult on present income |

Thank you. There are four more questions to go. They are a little bit more sensitive. If you would rather not answer you are free to do so.

Q24. In the last five years, have you had a close relative or friend diagnosed with a serious illness, for example cancer?

1 🞎 Yes 2 🞎 No

Q25. In the last five years, have you experienced the death of a close relative or friend?

1 🞎 Yes 2 🞎 No

Q26. In the last five years, have you been personally diagnosed with a serious illness, for example cancer?

1 🞎 Yes 2 🞎 No

Q27. Have you ever been involved in supporting and caring for a close relative or friend in their last few months of life?

1 🞎 Yes 2 🞎 No

Q28. Before we close is there anything else you would like to say? *(open-ended question)*
