# Supplementary material for: Choosing care homes as the least preferred place to die: a cross-national survey of public preferences in seven European countries
Source: BMC Palliat Care. 2014 Oct 23;13:48. doi: 10.1186/1472-684X-13-48 (PMC4430987; doi:10.1186/1472-684X-13-48)
Supplement: Supplementary file 2 — Additional file 2: Questions on preferences for place of death. (DOC 51 KB) [file 12904_2014_229_MOESM2_ESM.doc]

**Additional file 2**. Questions on preferences for place of death

| **England** | *In a situation of serious illness like cancer with less than one year to live....*   1. Where do you think you would prefer to die **if circumstances allowed you to choose**? I will now read out the answer options. 2. **So which of these do you think you would least prefer if circumstances allowed you to choose? I can read the list again if you would like me to.**  - **In your own home** - **In the home of a relative or friend** - **In a hospice or palliative care unit - places with specialised care and beds for dying patients** - **In hospital - but not in a palliative care unit** - **In a nursing home** - **In a residential home** - **Somewhere else: _______________** |
| --- | --- |
| **Flanders** | *Bij een ernstige ziekte bijvoorbeeld kanker, met nog minder dan een jaar te leven...*   1. Waar denkt u in dat geval, indien de omstandigheden u toelaten te kiezen, te willen sterven? Ik lees nu de keuzemogelijkheden voor. 2. **En waar zou u het minst graag sterven? Ik kan de lijst nog eens voorlezen, indien u dat wenst**.  - **In uw eigen huis** - **In het huis van een familielid of vriend** - **In een hospice of de palliatieve zorgafdeling van een ziekenhuis – plek met gespecialiseerde verzorging en bedden voor terminale patiënten** - **In een ziekenhuis – maar niet op de palliatieve zorgafdeling** - **In een residentieel woon- en zorgcentrum (vroeger rust- en verzorgingstehuis genoemd)** - **Elders (vermeld waar): __________________** |
| **Germany** | *In einer Situation mit einer schwerwiegenden Erkrankung, wie z.B. Krebs, und weniger als einem Jahr zu leben...*   1. Wo glauben Sie, würden Sie am liebsten sterben wollen, wenn die Umstände Ihnen eine Auswahl erlauben würden? Ich werde Ihnen nun die Antwortmöglichkeiten vorlesen. 2. **Und wo würden sie am wenigsten gern sterben wollen? Ich kann Ihnen die Liste noch einmal vorlesen, wenn Sie das wünschen.**  - **In Ihrem eigenen Zuhause** - **Im Haus eines Verwandten oder Freundes** - **In einem Hospiz oder auf einer Palliativstation – Orte mit spezialisierter Versorgung und Betten für sterbende Patienten** - **Im Krankenhaus – aber nicht auf einer Palliativstation** - **In einem Alten- oder Pflegeheim** - **Anderer Ort (bitte geben Sie an, wo): __________________** |
| **Italy** | *In una situazione di malattia grave come il cancro con meno di un anno da vivere...*   1. Dove pensa che Lei preferirebbe finire i suoi giorni, se le circostanze Le permettessero di scegliere? Ora Le leggerò le possibili risposte. 2. **E quale sarebbe l’ultimo posto dove vorrebbe morire? Se desidera, posso rileggerLe l’elenco.**  - **Elenco delle risposte** - **Nelle mia casa** - **In casa di un parente o di un amico** - **In un Hospice o in un reparto di Cure Palliative – un luogo di assistenza specialistica, con letti per i malati terminali.** - **In Ospedale – ma non in un reparto di Cure Palliative** - **In casa di riposo** - **Altrove (per favore specificare) _________________** |
| **Netherlands** | *In een situatie van ernstige ziekte, bijvoorbeeld kanker, en minder dan een jaar te leven...*   1. Waar denkt u dat u het liefst zou willen sterven, als de omstandigheden het toelaten te kiezen?Ik zal de antwoordmogelijkheden voorlezen. 2. **En waar zou u het minst graag sterven? Ik kan de lijst nog een keer voorlezen, als u dat wilt.**  - **Thuis** - **Bij een familielid of vriend thuis** - **In een hospice of palliatieve zorg unit – plaatsen met gespecialiseerde zorg en bedden voor stervende mensen** - **In het ziekenhuis – maar niet op een palliatieve zorg unit** - **In een verpleeghuis** - **In een verzorgingshuis** - **Ergens anders, namelijk: __________________** |
| **Portugal** | *Numa situação de doença grave, como o cancro, com menos de um ano de vida...*   1. Onde acha que preferiria morrer se as circunstâncias lhe permitissem escolher? Vou passar a ler-lhe as opções de resposta. 2. **E onde é que menos gostaria de morrer? Posso voltar a ler a lista, se quiser.**  - **Em sua casa** - **Em casa de um familiar ou amigo** - **Numa unidade de cuidados paliativos — locais com cuidados especializados e camas para doentes terminais.** - **No hospital – mas não numa unidade de cuidados paliativos** - **Num lar ou residência** - **Noutro sítio (diga qual, por favor): __________________** |
| **Spain** | *En una situación de enfermedad grave, como un cáncer, con menos de un año de vida...*   1. ¿dónde piensa que preferiría morir si las circunstancias le permitieran elegir? Ahora le leeré las posibles respuestas. 2. **¿Y cuál es el lugar en el que menos le gustaría morir? Le puedo leer la lista otra vez si Ud. quiere.**  - **En su propia casa** - **En casa de un familiar o amigo** - **En una unidad de cuidados paliativos – en un lugar con camas y cuidados especializados para pacientes que se están muriendo** - **En el hospital, pero no en una unidad de cuidados paliativos** - **En una residencia** - **En algún otro lugar (por favor indique dónde): __________** |

Note: In England and in the Netherlands both “nursing home” and “residential home” were available as separate answer options and later merged into “care homes”
