# Supplementary material for: Choosing care homes as the least preferred place to die: a cross-national survey of public preferences in seven European countries
Source: BMC Palliat Care. 2014 Oct 23;13:48. doi: 10.1186/1472-684X-13-48 (PMC4430987; doi:10.1186/1472-684X-13-48)
Supplement: Supplementary file 3 — Additional file 3: Variables tested in bivariate analysis. (DOC 35 KB) [file 12904_2014_229_MOESM3_ESM.doc]

**Additional file 3: Variables tested in bivariate analysis**

Bivariate analysis: tests and variables

| **Statistical test** | **Purpose, variables and corresponding question in questionnaire** |
| --- | --- |
| *χ2 test* | To compare the crude percentages of choosing care home as the least preferred place with crude percentages from those who chose elsewhere (‘care home’ versus ‘others’) according to:   - preferences for information (yes, always vs. all other options – variable recoded into binary) – questions 1,2 and 3 - concern with nine symptoms and problems at the end of life (top concern vs. others – variable recoded into binary) - question 4 - involvement of specific people in decisions in scenario of capacity and incapacity (yes/no) – questions 5 and 6 - priority attached to different goals at the end of life (top priority vs. others – variable recoded into binary) - questions 8 and 9 - gender (male/female) – question 12 - country of birth (born in country or born overseas) – question 13 - religion/denomination (yes/no) – question 17A - marital status (married/with partner, widowed, separated/divorced, single) – created from question 19 - living arrangements (alone or with others) – created from questions 20 and 21 - activities in last seven days (yes/no) – created from question 22 - person experience of close one with serious illness (yes/no) - question 24 - person experience of close one death (yes/no) – question 25 - person experience of serious illness (yes/no) – question 26 - person experience of caring for close one in last months of life (yes/no) – question 27 - Geographical region (NUTS levels 1 and 2) – not available in questionnaire, provided by market research company based on phone numbers |
| *Mann-Whitney test* | To compare those who chose care home as the least preferred place with  those who chose elsewhere (‘care home’ versus ‘others’) in terms of the  distribution in levels of:   - importance attributed to dying in the preferred place – question 10 - age bands - created from question 11 - area urbanisation – question 15 - education – question 16 - general health – question 18 - financial hardship – question 23 |
| *t-test* | To compare the mean age of those who chose care home as their least preferred place with those who chose elsewhere. Age was normally distributed across countries – question 11 (variable further recoded into binary in regression analysis) |
