# Supplementary material for: Choosing care homes as the least preferred place to die: a cross-national survey of public preferences in seven European countries
Source: BMC Palliat Care. 2014 Oct 23;13:48. doi: 10.1186/1472-684X-13-48 (PMC4430987; doi:10.1186/1472-684X-13-48)
Supplement: Supplementary file 4 — Additional file 4: Additional participant demographics and experiences of illness, death and dying by country. (DOCX 21 KB) [file 12904_2014_229_MOESM4_ESM.docx]

**Additional file 4: Additional participant demographics and experiences of illness, death and dying by country (N=9,344)**

| Variables^*, †^ | **England**  *N*=1,351 | **Flanders** *N*=1,269 | **Germany** *N*=1,363 | **Italy**  *N*=1,352 | **Netherlands**  *N*=1,356 | **Portugal**  *N*=1,286 | **Spain**  *N*=1,367 | **All countries**  *N*=9,344 |
| --- | --- | --- | --- | --- | --- | --- | --- | --- |
|  | n(%) | n(%) | n(%) | n(%) | n(%) | n(%) | n(%) | n(%) |
| **Additional demographics:** |  |  |  |  |  |  |  |  |
| Urbanisation level |  |  |  |  |  |  |  |  |
| Big city or suburbs/outskirts | 500 (37.1) | 289 (22.8) | 556 (40.9) | 269 (19.9) | 363 (26.8) | 643 (50.0) | 324 (23.7) | 2,944 (31.5) |
| Town or small city | 495 (36.7) | 224 (17.7) | 419 (30.8) | 526 (39.0) | 311 (23.0) | 368 (28.6) | 589 (43.1) | 2,932 (31.4) |
| Country village | 287 (21.3) | 591 (46.6) | 301 (22.1) | 521 (38.6) | 578 (42.7) | 221 (17.2) | 401 (29.4) | 2,900 (31.1) |
| Farm or home in countryside | 66 (4.9) | 165 (13.0) | 85 (6.2) | 34 (2.5) | 103 (7.6) | 54 (4.2) | 52 (3.8) | 559 (6.0) |
| Education level |  |  |  |  |  |  |  |  |
| Not completed primary education | 176 (13.8) | 26 (2.2) | 3 (0.2) | 5 (0.4) | 24 (1.8) | 18 (1.4) | 123 (9.1) | 375 (4.1) |
| Primary or first stage of basic | 3 (0.2) | 88 (7.3) | 27 (2.0) | 91 (6.9) | 30 (2.2) | 387 (30.2) | 195 (14.3) | 821 (9.0) |
| Lower secondary or second stage of basic | 338 (26.6) | 144 (11.9) | 634 (46.5) | 338 (25.6) | 333 (24.8) | 236 (18.4) | 327 (24.1) | 2,350 (25.7) |
| Upper secondary education | 204 (16.0) | 375 (31.0) | 172 (12.7) | 578 (43.7) | 458 (34.1) | 339 (26.4) | 248 (18.2) | 2,374 (26.0) |
| Post-secondary non-tertiary education | 35 (2.8) | 86 (7.1) | 516 (38.2) | 96 (7.3) | 59 (4.4) | 4 (0.3) | 53 (3.9) | 849 (9.3) |
| First stage tertiary education | 492 (38.7) | 479 (39.7) | 0 (0) | 189 (14.3) | 439 (32.7) | 292 (22.8) | 403 (29.7) | 2,294 (25.1) |
| Second stage tertiary education | 24 (1.9) | 10 (0.8) | 0 (0) | 25 (1.9) | 0 (0) | 7 (0.5) | 10 (0.7) | 76 (0.8) |
| Activities in last seven days ^‡^ |  |  |  |  |  |  |  |  |
| In paid work | 637 (47.3) | 649 (51.4) | 871 (64.4) | 590 (43.7) | 709 (52.6) | 561 (43.8) | 562 (41.3) | 4,579 (49.2) |
| In education | 88 (6.5) | 101 (8.0) | 178 (13.2) | 135 (10.0) | 80 (5.9) | 98 (7.7) | 155 (11.4) | 835 (9.0) |
| Retired | 480 (35.6) | 362 (28.7) | 289 (21.4) | 330 (24.5) | 342 (25.4) | 353 (27.6) | 245 (18.0) | 2,401 (25.8) |
| Unemployed | 63 (4.7) | 53 (4.2) | 65 (4.8) | 106 (7.9) | 48 (3.6) | 99 (7.7) | 185 (13.6) | 619 (6.7) |
| Permanently sick or disabled | 64 (4.7) | 46 (3.6) | 159 (11.8) | 12 (0.9) | 108 (8.0) | 38 (3.0) | 54 (4.0) | 481 (5.2) |
| Housework, looking after children or other persons | 389 (28.9) | 320 (25.3) | 477 (35.3) | 338 (25.1) | 372 (27.6) | 222 (17.3) | 378 (27.8) | 2,496 (26.8) |
| Financial hardship |  |  |  |  |  |  |  |  |
| Living comfortably on present income | 585 (43.8) | 689 (55.2) | 608 (45.5) | 430 (32.2) | 813 (60.9) | 222 (17.5) | 440 (32.6) | 3,787 (41.1) |
| Coping on present income | 576 (43.1) | 497 (39.8) | 618 (46.2) | 677 (50.7) | 441 (33.0) | 681 (53.6) | 633 (46.9) | 4,123 (44.8) |
| Difficult on present income | 136 (10.2) | 60 (4.8) | 85 (6.4) | 203 (15.2) | 62 (4.6) | 239 (18.8) | 203 (15.0) | 988 (10.7) |
| Very difficult on present income | 38 (2.8) | 3 (0.2) | 26 (1.9) | 25 (1.9) | 20 (1.5) | 129 (10.1) | 73 (5.4) | 314 (3.4) |
| Health |  |  |  |  |  |  |  |  |
| Very good | 565 (42.0) | 490 (38.6) | 310 (22.9) | 305 (22.6) | 302 (22.3) | 170 (13.3) | 293 (21.5) | 2,435 (26.1) |
| Good | 535 (39.8) | 583 (46.0) | 699 (51.5) | 642 (47.6) | 748 (55.3) | 488 (38.1) | 560 (41.1) | 4,255 (45.7) |
| Fair | 191 (14.2) | 176 (13.9) | 289 (21.3) | 377 (28.0) | 254 (18.8) | 558 (43.6) | 437 (32.0) | 2,282 (24.5) |
| Bad | 47 (3.5) | 16 (1.3) | 53 (3.9) | 21 (1.6) | 43 (3.2) | 46 (3.6) | 67 (4.9) | 293 (3.1) |
| Very bad | 7 (0.5) | 3 (0.2) | 5 (0.4) | 3 (0.2) | 5 (0.4) | 19 (1.5) | 7 (0.5) | 49 (0.5) |
| **Experience of serious illness, death and dying:** ^‡^ |  |  |  |  |  |  |  |  |
| Diagnosed with serious illness in last 5 years | 172 (12.8) | 190 (15.2) | 107 (8.0) | 113 (8.4) | 137 (10.1) | 99 (7.8) | 119 (8.8) | 937 (10.1) |
| Close relative/friend seriously ill in last 5 years | 849 (63.1) | 760 (60.6) | 862 (64.1) | 900 (67.4) | 969 (71.8) | 728 (57.5) | 923 (68.2) | 5,991 (64.8) |
| Death of close relative/friend in last 5 years | 949 (70.6) | 876 (69.9) | 933 (69.4) | 928 (69.3) | 1,036 (76.7) | 771 (60.9) | 1,006 (74.4) | 6,499 (70.3) |
| Cared for close relative/friend in last months of life | 679 (50.6) | 625 (49.9) | 647 (48.0) | 815 (60.8) | 702 (52.0) | 673 (53.2) | 771 (57.0) | 4,912 (53.1) |

^*^Sums may not always amount to the total sample number because of missing values on variables. Percentages may not always add up to 100 because of rounding. SD: standard deviation.

^†^The percentage of missing data was 0.1% for urbanisation level, 1.8% for education level, 0.4% for each activity in last seven days, 1.4% for financial hardship, 0.3% for health and 1.0% for each of the experiences of illness, death and dying. Missing data include “don’t know”, refusals, interview break-offs and data missing from the computer-assisted telephone interviewing (CATI) system.

^‡^Answers to these questions not mutually exclusive, each question on activities asked separately (answer options yes/no)
